# The limited diagnostic and prognostic utility of brief cognitive screening tools in acute stroke

**DOI:** 10.1093/esj/aakag047

**Published:** 2026-06-16

**Authors:** Rachael Gartly, Emma Elliott, Martin Taylor-Rowan, Bogna Drozdowska, Jodi Watt, Terence J Quinn

**Affiliations:** School of Cardiovascular & Metabolic Health, University of Glasgow, Glasgow, United Kingdom; School of Cardiovascular & Metabolic Health, University of Glasgow, Glasgow, United Kingdom; School of Health Sciences, University of Manchester, Manchester, United Kingdom; School of Health and Wellbeing, University of Glasgow, Glasgow, United Kingdom; Cumming School of Medicine, University of Calgary, Calgary, Alberta, Canada; School of Cardiovascular & Metabolic Health, University of Glasgow, Glasgow, United Kingdom; School of Cardiovascular & Metabolic Health, University of Glasgow, Glasgow, United Kingdom

**Keywords:** cognitive impairment, screening, sensitivity, specificity, stroke

## Abstract

**Introduction:**

Guidelines recommend cognitive screening post-stroke, but there is no consensus on approach. Given the dynamic nature of cognition following stroke, acute screening should both detect prevalent issues (diagnosis) and predict persisting problems (prognosis). We describe the diagnostic and prognostic utility of brief cognitive screening tools.

**Patients and methods:**

Patients were screened on admission with stroke using 12 modified screening tests: 10 and 4 question Abbreviated Mental Test, Cog-4, Clock Drawing test (CDT), Cognitive Impairment Test, informal bedside assessment, General Practitioner Assessment of Cognition, Minicog, Short Form Montreal Cognitive Assessment, Six-Item Screener (SIS), Harmonised Vascular Cognitive Impairment battery and 4-A’s Test. Sensitivity, specificity, positive predictive value (PPV) and negative predictive value were calculated against a Diagnostic and Statistical Manual of Mental Disorders, Fifth Edition adjudicated reference standard of neurocognitive disorders. Test accuracy was compared using area under the receiver operator characteristic curves.

**Results:**

Of 335 patients, 54 (16.1%) had pre-stroke neurocognitive disorder, 79 (23.6%) had 18-month neurocognitive disorder. Ten of 12 screening tests were more specific than sensitive. Informal bedside assessment had highest specificity (96%), but low sensitivity (9%); CDT had highest sensitivity (80%) but low specificity (33%). Negative predictive value ranged from 77% to 87%, PPV ranged from 27% to 54%. Area under the receiver operator characteristic curve ranged 0.53 (informal bedside assessment) to 0.69 (SIS).

**Discussion:**

In the acute setting, where the intention of screening is often to triage those who need further assessment, the pattern of high specificity at the expense of sensitivity is the opposite of what is desired.

**Conclusion:**

Brief cognitive screening tools, used in isolation, may not be suitable for assessment in acute stroke settings.

## Graphical Abstract

**Graphical Abstract:**
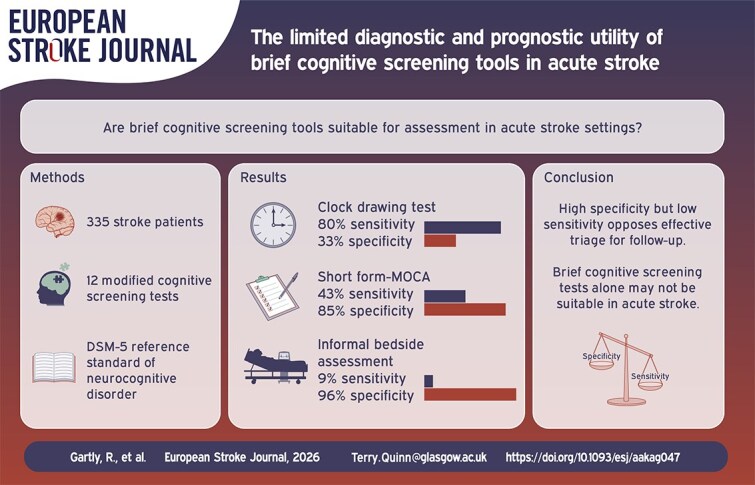

## Introduction

Cognitive impairment post-stroke is common.^1^ Estimates of cognitive impairment following stroke vary according to definition and timing. For the more standardised diagnosis of dementia, this is seen in 1 in 10 people pre-stroke and one in 5 stroke survivors.^2^ In the context of stroke, the patterns and natural history of cognitive impairment differ from those seen in classical neurodegenerative conditions such as Alzheimer’s disease.^3^ Cognitive issues following stroke may develop acutely or be an exacerbation of prevalent issues, problems may be transient or long-lasting,^3^ and impairments can be seen in any cognitive domain.^3^^,^^4^

The first step in managing cognitive problems is recognition. For this reason, National Clinical Guidelines for Stroke for the United Kingdom and Ireland,^5^ Canadian Stroke Best Practice Recommendations^4^ and other international guidance^6^ all recommend that cognitive screening should be carried out promptly following stroke. This is in the belief that early recognition of cognitive issues will assist in planning immediate care pathways, will identify those who need further assessment or modified interventions and will ultimately improve outcomes.^1^^,^^4^^,^^5^^,^^7^^,^^8^ While intuitive, there are no empirical data to support this assertion.^6^

The lack of evidence does not suggest lack of importance. European guidelines noted that cognition was a priority for future research in stroke care.^6^ Moreover, stroke survivors have highlighted the importance of cognition in national and international priority setting exercises.^9–11^ This emphasises the need for improved understanding of diagnosis and prognosis relating to cognition in acute stroke.

There are several cognitive screening tests potentially available for use in stroke, each encompassing different questions aimed at assessing varying cognitive domains.^12^ One systematic review identified 25 different tools,^13^ while a questionnaire-based survey found 45 different cognitive assessments used in practice.^14^ Overall, there is no consensus on which cognitive screening tool is superior.^6^^,^^12^^,^^13^^,^^15^^,^^16^ Cognitive screening in the context of stroke presents particular challenges around feasibility, acceptability and accuracy.^1^^,^^4^ Popular tools for cognitive assessment after stroke, such as the Montreal Cognitive Assessment (MoCA) and Mini Mental State Examination,^3^^,^^4^^,^^15^ are not suited to acute settings because they take time to administer and can be biased by non-cognitive stroke consequences, such as motor or visual field deficits.^13^^,^^17^

Brief screening tests, usually defined as taking less than 5 min to administer, could have particular applicability in the acute stroke setting.^13^ These short screening tools should minimise the time and resources needed for cognitive assessment and be easily performed by non-cognitive specialists with minimal need for training.^5^^,^^12^ A systematic review of test accuracy found no evidence that longer cognitive screening tools were better than brief screening tools.^13^ However, brief screening tools are not diagnostic in themselves, rather they can select stroke survivors who have cognitive issues that may require intervention (diagnostic) and triage those who are at risk of future cognitive problems and may require additional assessment and follow-up (prognostic).^5^

There are many potential benefits of cognitive screening in stroke, for example, profiling of stroke-related deficits to guide treatment formulations. Very brief screens designed to identify global impairment are not suited to this purpose. Rather, a suitable brief screening tool for use in acute stroke will both detect those people who have prevalent cognitive issues, and also suggest which stroke survivors are likely to have persisting issues beyond the initial period of dynamic cognitive change seen in the first weeks to months following stroke. If the purpose of testing is a combination of diagnosis of prevalent disease and prediction of incident disease, then arguably the ideal test would demonstrate high sensitivity, even if this comes at the cost of reduced specificity.

Most assessments of cognitive impairment in stroke have focussed on either diagnostic or prognostic intent, and have considered individual screening tools in isolation. We aimed to describe and compare both the diagnostic and prognostic utility of multiple brief (administrable in less than 5 min) cognitive screening tools when used in acute stroke services.

## Patients and methods

The APPLE (Assessing Post-Stroke Psychology Longitudinal Evaluation) study^18^ was designed to assess the diagnostic test accuracy of cognitive screening tools in acute stroke services. APPLE was approved by the Scotland A Research Ethics Committee, and local R&D approval was obtained for all participating sites (REC number: 16/SS/0105).^18^ This study has been reported in line with the Standards for Reporting Diagnostic test accuracy in dementia (STARDdem) guidances where relevant (Supplementary Materials).^19^ Study data are available through the Dementia Platforms UK Data Portal.

We approached suitable patients admitted with stroke, performed baseline assessments and then followed up to 18 months. Inclusion was by informed consent from the patient directly, or indirectly via a suitable proxy for those without capacity. Data collection was between October 2016 and January 2019, with differing services recruiting at differing times during this period. Eight acute stroke services in Scotland contributed data to the analysis presented here. Sites were spread across regions of Scotland, but all offered acute stroke services.

We aimed for an inclusive approach to recruitment, with the intention of mirroring clinical practice of those units that offer cognitive screening to all stroke admissions. The primary inclusion criterion was that the clinical team believed cognitive and mood testing was appropriate for the patient. Exclusions were minimal and related to practicalities (ability to complete screening in English) or legal mandates (no recruitment of prisoners). Further details including inclusion and exclusion criteria are described in the protocol paper.^18^

The ability to calculate sample sizes for future studies of test properties was an intended aim of APPLE^18^ and no a-priori sample size calculations were performed. However, based on a published sample size calculator, our recruited numbers were sufficient to support a test accuracy study with sensitivity and specificity between 65% and 95%, assuming a 10% marginal error; and to support a comparative study where differences range from 12% to 20%.^20^

The components of diagnostic test accuracy for the analyses were:

- Index tests: brief cognitive screening tests, suitable for “bedside” use, requiring minimal training, and taking less than 5 min to administer.
- Reference standard: adjudicated assessment for pre-stroke neurocognitive disorder (baseline diagnostic assessment), and adjudicated 18-month cognitive outcomes (prognostic).
- Condition of interest: cognitive impairment.
- Setting: hospitals in Scotland with acute stroke services.

There is no “gold standard” approach to assessment for studies of cognitive impairment in stroke and the condition is variably defined in the literature. For this project, “cognitive impairment” refers to a clinical cognitive syndrome encompassing both minor (equivalent to mild cognitive impairment) and major neurocognitive disorders (equivalent to dementia syndrome) based on the Diagnostic and Statistical Manual of Mental Disorders, Fifth Edition (DSM-5).^21^ Participants were categorised as having: (i) no cognitive impairment, (ii) a minor neurocognitive disorder or (iii) a major neurocognitive disorder. We did not attempt to formulate alternate diagnoses. The screening was used for research purposes only and so did not influence care.

## Data collection

### Brief cognitive screening tests

The baseline assessment of cognitive status was completed within the first days of stroke admission, with site investigators instructed to assess patients as soon as possible. The assessment battery^18^ involved a composite of questions comprising items from popular brief cognitive screening tools.

Choice of tests for inclusion was based on prior use in stroke or other acute settings, familiarity in UK secondary care, and administration time of < 5 min. Including unique component test items in a single battery allowed for derivation of multiple tests while avoiding repetition of individual questions, thus minimising patient test burden. Reference standard results were not available to the researchers administering the screening tools.

The brief tests evaluated were modified versions of the: 10-point Abbreviated Mental Test^22^; 4-item Abbreviated Mental Test (4-AMT)^23^; 4-A’s Test^24^; 6-item Cognitive Impairment Test (6-CIT)^25^; Clock Drawing Test (CDT); an informal bedside assessment (unscripted short assessment by clinicians)^26^; General Practitioner Assessment of Cognition (GP-Cog)^26^; Cog-4 derived from the National Institutes of Health Stroke Scale (NIHSS)^27^; Short-form Montreal Cognitive Assessment (Short Form MoCA)^28^; Six-Item Screener (SIS)^29^; Harmonised Vascular Cognitive Impairment battery (VCI-H)^12^ and the Minicog.^30^ The content of the informal bedside assessment was at the tester’s discretion and was taken from clinician assessment for “confusion” that formed part of the daily assessment in all participating sites.

We derived 10 of the brief cognitive screening tests^12^^,^^22–26^^,^^28–31^ from our test battery using varying question combinations (Supplementary Materials). For example, for the 4-AMT,^23^ patients received a mark for correctly answering their date of birth, age, present year and place from the longer list of questions and tasks. Certain aspects of component test items were modified to allow derivation of the maximum number of brief tests, for example, where screening tools used recall tasks, a single set of words was used to avoid repetition (Supplementary Materials). Operationalisation of all scoring is fully described in the Supplementary Materials.

Patients received a score of zero for questions which were incomplete due to aphasia, motor problems or deafness. Tests were not adapted to accommodate for these impairments. Patients were classified as cognitively impaired on screening using a binary outcome (yes/no) based on pre-specified cut-offs as per published standards for each screening tool (Table 1).^12^^,^^22–26^^,^^28–31^ One screening test, the 6-CIT, offers different cut-offs to differentiate mild and severe cognitive impairment, and the threshold for the more severe form was chosen. Best practice recommends this approach to adjusting the threshold used in the acute stroke period when cognition is unstable.^6^

**Table 1:** Pre-specified cut-offs used to classify patients as cognitively impaired, as per published standards.^12^^,^^22–26^^,^^28–31^ Using these, the number of participants of the baseline population who were identified as cognitively impaired is shown.

Cognitive screening test	Maximum possible score	Cut-off score for cognitive impairment	Number of participants identified as cognitively impaired post-stroke	
			Participants with pre-stroke cognitive impairment (*n* = 54)	All participants (Total *n* = 335)
**10-AMT**	10	<7	16 (30%)	41 (12%)
**4-AMT**	4	<4	20 (37%)	48 (14%)
**GP-Cog**	9	<5	29 (54%)	90 (27%)
**Minicog**	5	<3	27 (50%)	86 (26%)
**6-CIT**	28	>9	26 (48%)	69 (21%)
**VCI-H**	11	<9	41 (76%)	151 (45%)
**SF-MoCA**	8	<4	24 (44%)	73 (22%)
**4AT**	4	>1	13 (24%)	36 (11%)
**SIS**	6	<5	30 (56%)	80 (24%)
**Informal**	5	<5	4 (7%)	17 (5%)
**Cog-4**	9	>0	17 (31%)	76 (23%)
**CDT**	3	<3	39 (72%)	235 (70%)

Abbreviations: 4-AMT = 4-item Abbreviated Mental Test; 10-AMT = 10-item Abbreviated Mental Test; 4AT = 4-As Test; CDT = Clock Drawing Test; 6-CIT = 6-item Cognitive Impairment Test; GP-Cog = General Practitioner Assessment of Cognition; SF-MoCA = Short Form Montreal Cognitive Assessment; SIS = Six Item Screener; VCI-H = Harmonised Vascular Cognitive Impairment battery.

### Adjudicated assessment for pre-stroke and 18-month cognitive outcomes

In the first weeks following recruitment, participants, and, where applicable, a suitable informant, were assessed for pre-stroke neurocognitive disorder. A trained researcher, independent of the main data collection, performed a structured clinical interview based on the Clinical Dementia Rating Scale.^31^ We used this assessment as a reference standard for the brief screening tests. Our intention here was not to describe the properties of the screeners for diagnosing pre-stroke problems (as this would be confounded by post stroke cognitive issues), but rather we assumed that all those with pre-stroke cognitive issues would still have cognitive issues at time of acute assessment, and so these data allowed for a validity check of specificity and related metrics.

Outcomes at 18 months were formulated using a similar approach, incorporating the previous structured interview, multidomain neuropsychological testing performed at regular intervals as part of the study design, neuroimaging, laboratory results and participants’ medical records.^18^

In both instances, the assessment and related data were adjudicated by a physician and psychologist with expertise in stroke cognitive assessment to make a final consensus formulation based on DSM-5 criteria.^21^

We did not perform inter- or intra-rater agreement reliability studies. The reference standard was not based on the results of the cognitive screening, but assessors had access to results of all cognitive assessments available. Clinical interventions between index test administration and reference standard adjudication constituted routine clinical care in line with guidelines.

As an internal validity check of the 18-month cognitive outcomes, data from general cognitive assessments performed at 12 and 18 months were also assessed. For patients who had both 12- and 18-month evaluations available, only their 18-month score was used. For consistency, we used data from participants who completed either the MoCA^6^ or Modified Telephone Interview for Cognitive Status (TICS-m).^32^ Montreal Cognitive Assessment used a stroke adjusted cut-off of < 22.^6^ Modified Telephone Interview for Cognitive Status was scored out of 39 using a cut-off of < 24.^32^ As before, participants were classed as cognitively impaired if they were untestable, for example, due to aphasia. Participants who were classified as too unwell, who did not have enough time or who had another, non-medical reason listed for non-completion of tests were removed from this sensitivity analysis as their cognitive status was uncertain.

### Data analysis

We described the population using standard metrics. Using brief screening tools as our index tests of interest and adjudicated outcomes as our reference standard, we calculated sensitivity, specificity, positive (PPV) and negative predictive values (NPV) and plotted tests in receiver operating characteristic (ROC) space.

We calculated area under the ROC curve (AUROC) for each test. An AUROC value greater than 0.5 indicates the test’s discriminatory ability is better than that obtained by chance, and 0.7–0.8 is reported as acceptable.^33^ The differences between AUROCs were described to allow for comparative testing based on the DeLong method.^34^

Multiplicity of testing was accounted for by taking a threshold significance of *P* < .001. Initial analyses used SPSS (Version 28.0.0.0 (190), IBM, Armonk, NY). All values were cross-checked and 95% CIs obtained using MedCalc Software (Version 20.218, Ostend, Belgium). Scores on the brief screening tests were collated, tabulated and correlated with each other. GraphPad Prism (Version 9.4.1. (681), San Diego, CA, USA) was used to generate a colour map of correlation between each of the cognitive screening tests.

## Results

### Baseline characteristics

Three-hundred and thirty-five patients were recruited. Mean age at time of stroke was 69.6 years (SD: 12.7), 185 (55.2%) were female and median NIHSS score was 2 (25th–75th IQR: 1–4). One-hundred and fourteen (34.0%) patients were diagnosed with a partial anterior circulation stroke (Table 2). Patients were assessed with the brief screening tools at a median of 6 days post-stroke (25th–75th IQR: 4–9). The number of participants identified as cognitively impaired on each test is described in Table 1.

**Table 2:** Baseline characteristics.

Characteristic	Number (Total included *n* = 335)
**Age, years (mean, SD)**	69.6 (12.7)
**Sex, female (%)**	185 (55.2)
**NIHSS (median, IQR)**	2 (1–4)
**Pre-stroke Barthel Index (median, IQR)**	18.9 (18–20)
**Pre-stroke mRS (median, IQR)**	1 (0–2)
**Stroke classification (%)**	
**TACS**	26 (7.8)
**PACS**	114 (34.0)
**LACS**	82 (24.5)
**POCS**	66 (19.7)
**Other/unclassified**	47 (14.0)
**Pre-stroke comorbidities (%)**	
**Atrial fibrillation**	55 (16.4%)
**Diabetes mellitus**	81 (24.2%)
**Previous stroke/TIA**	85 (25.4%)
**Sensory impairment**	132 (39%)
**Depressive symptoms**	43 (13%)
**Diagnosis of depression**	98 (29%)
**Problematic alcohol or illicit drug use**	40 (12%)
**First degree relative with dementia**	49 (15%)

Abbreviations: TACS = total anterior circulation stroke; PACS = partial anterior circulation stroke; LACS = lacunar stroke; POCS = posterior circulation stroke.

“Depressive symptoms” label signifies active depression symptoms in months before stroke.

### Adjudicated assessment for pre-stroke neurocognitive disorder

Fifty-four (16.1%) participants were identified as having neurocognitive disorder pre-stroke (diagnostic) (Table 1). This group was best identified using the VCI-H (*n* = 41, sensitivity 76% [95% CI, 62–87]), while an informal bedside assessment picked up the fewest number of cases (*n* = 4, sensitivity 7% [95% CI, 2–18]) (Table 3).

**Table 3:** Test accuracy metrics for the brief baseline cognitive screening tests using the adjudicated pre-stroke and 18-month neurocognitive outcomes (*n* = 335).

Cognitive screening test	Pre-stroke (Prevalence 16.1%)		18 Months (Prevalence 23.6%)			
	Sensitivity % [95% CI]	PPV % [95% CI]	Sensitivity % [95% CI]	Specificity % [95% CI]	PPV % [95% CI]	NPV % [95% CI]
**10-AMT**	30 [18–44]	39 [27–53]	27 [17–38]	92 [88–95]	51 [38–65]	80 [78–82]
**4-AMT**	37 [24–51]	42 [30–54]	32 [23–44]	91 [87–95]	54 [42–66]	82 [79–84]
**GP-Cog**	54 [40–67]	32 [25–40]	54 [43–66]	82 [76–86]	48 [40–56]	85 [82–88]
**Minicog**	50 [36–64]	31 [24–39]	49 [38–61]	82 [76–86]	45 [37–54]	84 [81–87]
**6-CIT**	48 [34–62]	38 [29–47]	44 [33–56]	87 [82–91]	51 [41–61]	83 [80–86]
**VCI-H**	76 [62–87]	27 [23–31]	71 [60–81]	63 [57–69]	37 [32–42]	87 [83–91]
**SF-MoCA**	44 [31–59]	33 [25–42]	43 [32–55]	85 [80–89]	47 [37–56]	83 [80–85]
**4AT**	24 [13–38]	36 [23–51]	24 [15–35]	93 [90–96]	53 [38–67]	80 [78–82]
**SIS**	56 [41–69]	38 [30–46]	53 [42–64]	85 [80–89]	53 [44–61]	85 [82–88]
**Informal**	7 [2–18]	24 [9–48]	9 [4–17]	96 [93–98]	41 [22–64]	77 [76–79]
**Cog-4**	32 [20–46]	22 [15–31]	35 [25–47]	81 [76–86]	37 [28–46]	80 [77–83]
**CDT**	72 [58–84]	17 [14–19]	80 [69–88]	33 [27–39]	27 [24–30]	84 [77–89]

Abbreviations: 4-AMT = 4-item Abbreviated Mental Test; 10-AMT = 10-item Abbreviated Mental Test; 4AT = 4-As Test; CDT = Clock Drawing Test; 6-CIT = 6-item Cognitive Impairment Test; GP-Cog = General Practitioner Assessment of Cognition; NPV = negative predictive value; PPV = positive predictive value; SF-MoCA = Short Form Montreal Cognitive Assessment; SIS = Six Item Screener; VCI-H = Harmonised Vascular Cognitive Impairment battery.

### Adjudicated 18-month neurocognitive disorder

Based on 18-month adjudicated cognitive outcomes, the overall prevalence of any neurocognitive disorder was 23.6% (*n* = 79). This included all patients diagnosed with pre-stroke neurocognitive disorder. Ten of the 12 brief cognitive screening tests were more specific than they were sensitive (Table 3). Informal bedside assessment generated the highest specificity of 96% (95% CI, 93–98), but sensitivity was low at 9% (95% CI, 4–17) compared with a high sensitivity of 80% (95% CI, 69–88) for the CDT.

Accounting for the cohort population prevalence, the NPV ranged from 77% for an informal bedside assessment to 87% for the VCI-H. The chance of truly having cognitive impairment at 18 months post-stroke, when indicated by a baseline test, was highest for the 4-AMT with a PPV of 54% (Table 3).

**Figure 1:**
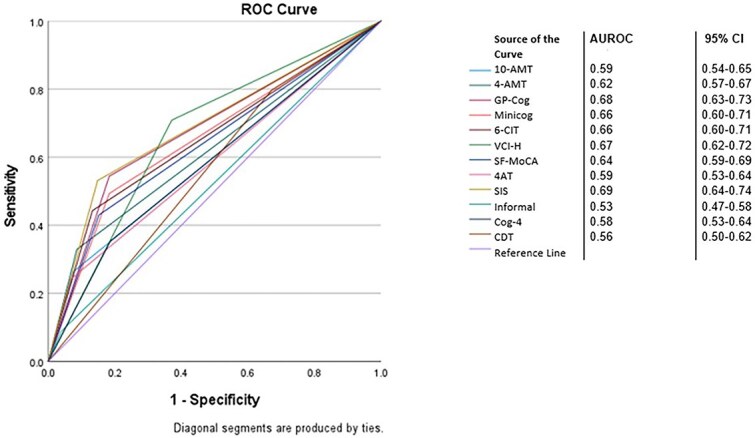
Receiver operator characteristic curves for the brief cognitive screening tests assessed. See corresponding key for the source of the curve.

The AUROC for 18-month outcomes ranged from 0.53 (95% CI, 0.47–0.58) for an informal bedside assessment to 0.69 (95% CI, 0.64–0.74) for the SIS (Figure 1). Differences between tests are described in the Supplementary Materials. The largest difference was 0.17 between the SIS and informal bedside assessment.

Data from 104 participants were available for the validation analysis using the MoCA and TICS-m, of whom 50 (48.1%) were classed as cognitively impaired. Eleven of the 12 tests were more specific than sensitive. The CDT remained the most sensitive at 74% with specificity of 37%. An informal bedside assessment was the least sensitive at 6% but the most specific at 98% (Table 4).

**Table 4:** Test accuracy metrics for the brief baseline cognitive screening tests against a reference standard of the longer 12- and 18-month multidomain neuropsychological assessments.

Cognitive screening test	Baseline cognitive screening tests compared to multidomain neuropsychological assessments			
	12 and 18 months combined (*n* = 104, prevalence 48.1%)			
	Sensitivity % [95% CI]	Specificity % [95% CI]	PPV % [95% CI]	NPV % [95% CI]
**10-AMT**	16 [7–29]	96 [87–100]	80 [47–95]	55 [52–59]
**4-AMT**	20 [10–34]	96 [87–100]	83 [54–96]	57 [53–60]
**GP-Cog**	40 [26–55]	87 [75–95]	74 [57–86]	61 [55–67]
**Minicog**	40 [26–55]	85 [73–93]	71 [55–84]	61 [54–66]
**6-CIT**	34 [21–49]	91 [80–97]	77 [58–90]	60 [54–65]
**VCI-H**	64 [49–77]	81 [69–91]	76 [64–85]	71 [62–78]
**SF-MoCA**	32 [20–47]	93 [82–98]	80 [59–92]	60 [54–64]
**4AT**	16 [7–29]	96 [87–100]	80 [47–95]	55 [52–59]
**SIS**	34 [21–49]	91 [80–97]	77 [58–90]	60 [54–65]
**Informal**	6 [1–17]	98 [90–100]	75 [24–97]	53 [51–55]
**Cog-4**	24 [13–38]	83 [71–92]	57 [38–74]	54 [49–59]
**CDT**	74 [60–85]	37 [24–51]	52 [46–59]	61 [46–73]

Abbreviations: 4-AMT = 4-item Abbreviated Mental Test; 10-AMT = 10-item Abbreviated Mental Test; 4AT = 4-As Test; CDT = Clock Drawing Test; 6-CIT = 6-item Cognitive Impairment Test; GP-Cog = General Practitioner Assessment of Cognition; NPV = negative predictive value; PPV = positive predictive value; SF-MoCA = Short Form Montreal Cognitive Assessment; SIS = Six Item Screener; VCI-H = Harmonised Vascular Cognitive Impairment battery.

### Correlation between cognitive screening tests

**Figure 2:**
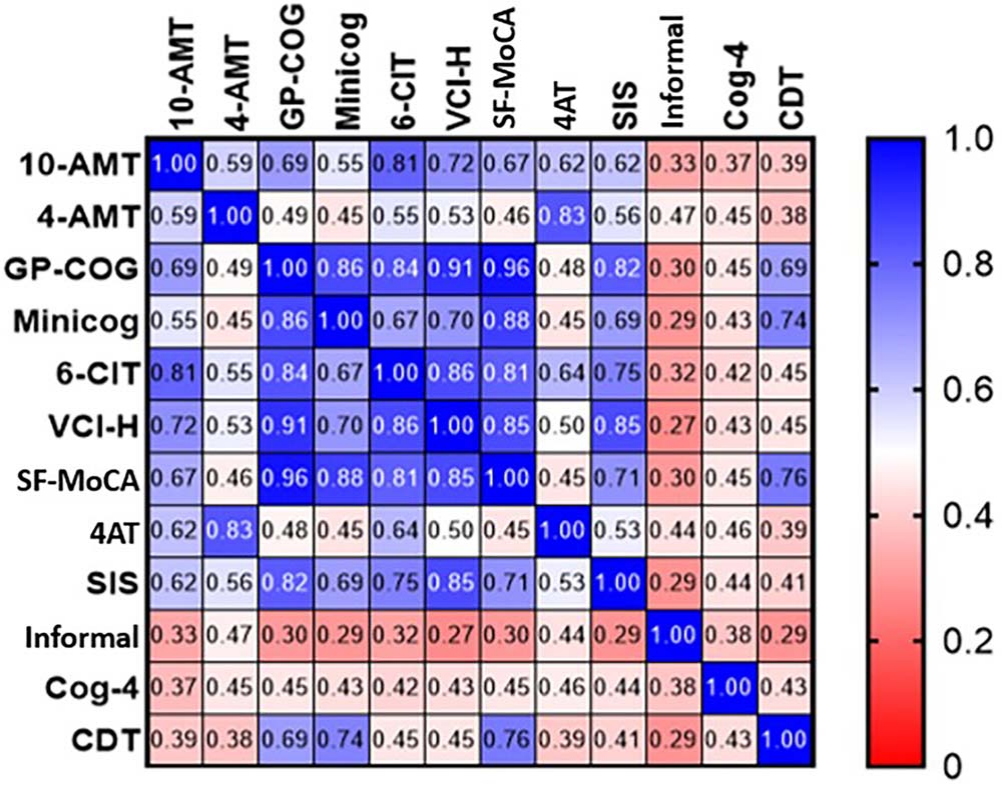
Heat map with numerical values representing the degree of correlation between each of the brief cognitive screening tests assessed. Values closer to one indicate a higher degree of correlation between tests (in blue).

The GP-Cog and Abbreviated Short Form MoCA were most highly correlated overall (0.96). Informal bedside assessments, Cog-4 and CDT demonstrated low correlation across tests, with lowest correlation between an informal bedside assessment and VCI-H (0.27) (Figure 2).

## Discussion

The primary aim of this study was to describe the diagnostic and prognostic utility of brief cognitive screening tools when used in acute stroke services. We found that many of these tests had poor sensitivity and thus risk missing people with cognitive issues. In the acute stroke setting, where the intention of screening is often to triage those who need further assessment, this pattern of high specificity and low sensitivity is the opposite of what is desired.

To put these data into context, based on a 30% prevalence of post-stroke cognitive impairment,^1^ a stroke unit admitting 1000 strokes per annum using an informal bedside assessment alone as the initial cognitive screen (sensitivity 9%, specificity 96%) would miss 273 people with cognitive problems and falsely label 28 people with cognitive impairment. On the other hand, using only the CDT (one of the best performing screening tools in this study with predictive sensitivity 80%, specificity 33%) would miss 60 people with cognitive problems and label 469 people with no problems as having cognitive impairment. While neither false negatives nor false positives are desirable, the consequences of not identifying an individual’s post-stroke cognitive impairment are arguably greater than the consequences of being falsely labelled and then requiring a more detailed cognitive assessment later in the admission.

We anticipated that all those with a clinical diagnosis of pre-stroke cognitive impairment would be picked up on post-stroke screening. Our analysis proved otherwise, with 7 of the 12 tests identifying less than 50% of participants with pre-stroke cognitive impairment. This highlights that other investigations should be performed in addition to brief screening tests to fully characterise the pre-stroke cognitive state as the screeners are not, in themselves, diagnostic.^35^

Although our data question their suitability in stroke, brief cognitive screening instruments have demonstrated useful properties in other healthcare settings. Systematic reviews exploring the test accuracy of the CDT and Minicog in community-dwelling older adults, report sensitivity of 75% or more, with specificity of 80% or higher to detect dementia.^36^ A potential reason for the differing test properties could be the non-acute nature of the assessment. There are multiple competing time and attentional demands on both the patient and the clinician in a busy acute stroke setting that could complicate assessment.

However, brief screening tools have demonstrated utility in certain acute settings. In the emergency department, the SIS has 94% sensitivity and 86% specificity to detect cognitive impairment, with equivalent figures for the Minicog.^37^ Also in the emergency department, a recent meta-analysis produced a summary estimate of 89% sensitivity and 67% specificity for the 6-CIT, while the SIS had 72% sensitivity and 79% specificity to detect cognitive impairment.^38^ This suggests that these tests can have utility in acute situations, and there may be specific issues relating to stroke that impact on test accuracy. Stroke-related physical, sensory and the dynamic nature of cognitive changes all further complicate the assessment process.

Our results of brief cognitive screening instruments showing greater specificity than sensitivity in the acute setting are in keeping with previous studies in stroke. An acute study testing brief screening tools against contemporaneous MoCA as a reference standard,^16^ obtained 100% specificity for certain tests but with poor sensitivity.^16^

### Strengths and limitations

Our study provides novel data around test accuracy that has immediate clinical applicability. Our focus on brief screening tools and acute settings speaks to a research-practice disconnect, as traditional studies of cognitive assessment in stroke have focussed on detailed batteries applied later in stroke recovery which are not transferable to the acute space. Our reference standard was robust, based on adjudication of multimodal data including laboratory values, imaging and neuropsychological testing.

The population included is generalisable to other unselected acute stroke populations, with age, sex and stroke severity of the study cohort generally in keeping with data from national audit in the United Kingdom.^39^ The stroke severity (NIHSS) seen in our population is lower than data for unselected hyper-acute stroke cohorts. However, our assessments took place in the first days, rather than first hours, and there will have been a degree of stroke recovery. This acute (days) rather than hyperacute (hours) screening is reflective of how these brief screens are used in practice. Only those patients where the clinical team felt screening was appropriate were included, and this will also have led to a reduced average NIHSS as the most severe strokes may not have been included. Again, this is reflective of clinical practice, where patients with reduced consciousness or requiring neuro-intensive care, are not assessed with brief cognitive and mood screens.

We followed best practice in conduct and reporting of test accuracy research,^19^ and our design allowed for head-to-head testing across multiple screening tools. We hope that these data will be more useful to clinicians, researchers and policy makers than previous studies which have assessed tools in isolation and have relied on indirect assessments of comparative efficacy.

We acknowledge certain potential biases inherent in our approach. There was a trade-off between test burden and fidelity to the original brief screening tool, this necessitated some changes to the items in certain tests (eg, delayed recall), and the order the items were administered. Despite instructions to perform cognitive screening as soon as possible, the timing of baseline assessments varied by several days between participants. This reflects the difficulty in performing acute cognitive assessment. Incorporation bias is possible as test components that are part of our brief screening tools could also be used as part of the diagnostic assessment and formulation. This is somewhat unavoidable due to the overlapping methods used to both screen and diagnose cognitive impairment. If anything, incorporation bias would improve test accuracy, so accuracy in practice may be even poorer than described here. We grouped minor and major neurocognitive disorder as one entity, but we acknowledge the major clinical differences between these syndromes.

Test non-completion rates were collected. While we attempted to describe reasons for non-completion, we could not fully differentiate non-completion due to cognitive issues from non-completion due to other issues, particularly since more than 1 factor may explain non-completion. Where there was non-completion, a score of “zero” was given. There is no consensus on how to handle missing cognitive data at a test, or individual question, level.^40^ Differing approaches can give varying overall results. The issues around non-completion in the APPLE cohort have been previously described.^41^

### Clinical and research implications

The immediate clinical implication of these data is that brief screening tests designed and validated in non-stroke settings may not be suitable early after stroke. If the intention of acute screening is to identify those people with prevalent or incident cognitive issues that require additional assessment, then the commonly used tests are not fit for purpose. When informal assessment of cognition is used, almost all detected are likely to have cognitive problems. However, this approach misses so many cases it should not be used in isolation. The clock draw may have merit in the acute setting as it offers greatest sensitivity, but it remains imperfect and could generate substantial additional work due to false positives. Combinations of tests used in parallel or in sequence could offer a solution, but this would need to be empirically tested for both test accuracy and feasibility.

We have assessed our data through the lens of using screening to identify those who may need further cognitive assessment, ie, assessing for global impairments. There are other rationales for cognitive screening in the stroke context, including identification of focal deficits to guide intervention, ie, assessing for domain-specific impairments. The tools used here are suited to the first intention, global cognitive assessment. Tools designed for domain specific impairments, that are suitable for use in acute stroke, have been developed and we would encourage their use.^42^

As well as research on the tests themselves, research around the timing and purpose of acute cognitive screening seems warranted. Teams should look to develop bespoke screening tests designed for the acute stroke setting. Such tests should focus on improving test feasibility and acceptability, for example, by using visual cues or non-verbal responses. Any new cognitive screening tools for use in stroke should be validated in this population, as extrapolating test accuracy from community or other non-acute studies is not valid.

## Conclusion

In the acute setting, where the intention of screening is often to triage those who need further assessment, the pattern of high specificity at the expense of sensitivity is the opposite of what is desired. Traditional brief cognitive screening tools, used in isolation, may not be suitable for assessment in acute stroke settings.

## Supplementary Material

Supplementary_materials_aakag047

## Data Availability

Data have been stored in the Virtual International Stroke Trials Archive (Cognition theme) VISTA-Cognition database and are also available through the MRC Dementia Platforms UK (DPUK) Data Portal.
