# Supplementary material for: The limited diagnostic and prognostic utility of brief cognitive screening tools in acute stroke
Source: Eur Stroke J. 2026 Jun 16;11(6):aakag047. doi: 10.1093/esj/aakag047 (PMC13271418; doi:10.1093/esj/aakag047)
Supplement: Supplementary_materials_aakag047 [file supplementary_materials_aakag047.zip › Supplementary Material revised clean version.docx]

**Supplementary Material**

**Contents**

Abbreviations………………………………………………………………………………………….2

STARDdem checklist………………………………………………………………………………3-5

Baseline cognitive assessment (AMT-plus) form……………………………………………….6-7

Index tests……………………………………………………………………………………………..8

Test score rules…………………………………………………………………………….….....9-10

Participants removed from the analysis…………………………………………………………..11

Cross-tabulation of index test and reference standard………………………………………….12

Table of the differences between the AUROC…………………………………………...………13

**Supplementary Material 1**

**Abbreviations**

| 10-AMT | 10-point Abbreviated Mental Test |
| --- | --- |
| 4-AMT | 4-item Abbreviated Mental Test |
| 4AT | 4 ‘A’s Test |
| 6-CIT | 6-item Cognitive Impairment Test |
| APPLE | Assessing Post-Stroke Psychology Longitudinal Evaluation |
| AUROC | Area Under the Receiver Operating Characteristic curve |
| CDT | Clock Drawing Test |
| CI | Confidence Interval |
| DSM-5 | Diagnostic and Statistical Manual of Mental Disorders, Fifth Edition |
| GP-Cog | General Practitioner Assessment of Cognition |
| Informal | Informal Bedside Assessment |
| MMSE | Mini-Mental State Examination |
| MoCA | Montreal Cognitive Assessment |
| NIHSS | National Institutes of Health Stroke Scale |
| NPV | Negative Predictive Value |
| PPV | Positive Predictive Value |
| SF-MoCA | Abbreviated Short-form Montreal Cognitive Assessment |
| SIS | Six-Item Screener |
| TIA | Transient Ischaemic Attack |
| TICS-m | Modified Telephone Interview for Cognitive Status |
| VCI-H | Harmonised Vascular Cognitive Impairment battery |

**Supplementary Material 2**

**STARDdem Checklist**

**Table_e-1_print_version_of_STARDdem_table**

| **Section and Topic and item No.** | | **STARD checklist item** | **Points of particular relevance to dementia** | **Page** |
| --- | --- | --- | --- | --- |
| **Title/Abstract/**  **Keywords** |  | |  |  |
| 1 | | Identify the article as a study of diagnostic accuracy (recommend MeSH heading 'sensitivity and specificity') | Studies reporting a sensitivity/specificity or 2x2 data derivable, fall within the scope of STARDdem and should be indexed accordingly. | 1-2 |
| **Introduction** | |  |  |  |
| 2 | | State the research questions or study aims, such as estimating diagnostic accuracy or comparing accuracy between tests or across participant groups | Some studies describing aims related to 'prognosis' or 'prediction' may also fall within the remit of STARDdem.    Report test purpose: 'stand-alone' test or as an addition to other tests or clinical criteria. | 3-4 |
| **Methods** | |  |  |  |
| *Participants:* | |  |  |  |
| 3 | | The study population: The inclusion and exclusion criteria, setting and locations where data were collected  See also **Item 4** on recruitment and **Item 5** on sampling | Key inclusion criteria: (a) demographic, especially age; (b) cognition- or disease-related criteria. Accurate description of the target sample is required including reporting criteria used to define the study population.  Report referral pathways, precise locations of patient recruitment, where index test and reference standard were performed. For secondary/tertiary settings helpful to report the medical subspecialty or hospital dept (e.g. psychiatry, neurology).  Diagnostic accuracy studies in dementia are often nested within larger cohort studies. If this is the case, then the targeted population for the cohort study and the method of selection into the cohort should be described and/or the parent study cited. | 4-5 |
| 4 | | Participant recruitment: Was recruitment based on presenting symptoms, results from previous tests, or the fact that the participants had received the index tests or the reference standard?  See also **Item 5** on sampling and **Item 16** on participant loss at each stage of the study | Report whether those in intermediate categories (e.g. possible AD or possible DLB) were excluded. | 4-5 |
| 5 | | Participant sampling: Was the study population a consecutive series of participants defined by the selection criteria in item 3 and 4? If not, specify how participants were further selected  See also **Item 4** on recruitment and **Item 16** on participant loss | Planned analyses showing how characteristics of the subgroup entering the study differ from the eligible population are strongly recommended (i.e. if a convenience sample has been used due to invasive nature of test/s). | 4-5 |
| 6 | | Data collection: Was data collection planned before the index test and reference standard were performed (prospective study) or after (retrospective study)? | Authors should report the timing of the analysis plan with respect to data collection: was the analysis plan set out in a protocol before index and reference standards were performed? If not, when was the analysis plan created? | 4-5 |
| *Test methods:* | |  |  |  |
| 7 | | The reference standard and its rationale | For neuropathological and clinical reference standards the diagnostic criteria used should be specified. Where relevant, reference should be made to studies validating the criteria.  Report if standard consensus clinical criteria incorporate the index test (incorporation bias rendering blinding of index test impossible). | 5 |
| 8 | | Technical specifications of material and methods involved including how and when measurements were taken, and/or cite references for index tests and reference standard  See also **Item 10** concerning the person(s) executing the tests | *Use of scales*: specify details of administration, which version.  *Clinical diagnostic criteria*: what information was available to inform the diagnoses; how the criteria were applied (e.g. by individual clinicians, by consensus conference, by semi-automated algorithm).    *Imaging and laboratory tests*: specify materials and instruments, including sample handling and concordance with any harmonisation criteria. In new assays describe all steps in detail. Any particular preparation of participants should be described. | 5-7 |
| 9 | | Definition of and rationale for the units, cut-offs and/or categories of the results of the index tests and the reference standard | Justify any cut-offs used, as these may vary with clinical context. | 5-7 |
| 10 | | The number, training and expertise of the persons executing and reading the index tests and the reference standard  See also **Item 8** | Especially where subjective judgments are involved, e.g. the interpretation of neuroimaging results.  Report inter- and intra-rater agreement.  Reference or describe the content of training materials used.  Reference or describe details of lab certification and harmonised biomarker assays. | 5-7 |
| 11 | | Whether or not the readers of the index tests and reference standard were blind (masked) to the results of the other test and describe any other clinical information available to the readers  See also **Item 7** | Also, the index test may form a part of the reference standard. This is often referred to as incorporation bias and renders blinding of the index test impossible. | 6-7 |
| *Statistical methods:* | |  |  |  |
| 12 | | Methods for calculating or comparing measures of diagnostic accuracy, and the statistical methods used to quantify uncertainty (e.g. 95% confidence intervals) |  | 7-8 |
| 13 | | Methods for calculating test reproducibility, if done | Applies to the reference standard as well as to the index test. Both should be reported/adequately referenced. Report inter-rater and test-retest reliability of reference standard as established in the study being reported, rather than simply referring to other studies where reproducibility has been established.  The training which image readers receive should be carefully described. Studies in which the accuracy of ‘majority’ judgements are reported should also report data for the minority judgements. Reports of the impact of training should clearly describe the characteristics of the sample used for training and whether it is representative of the group to which the test will be applied. | 7-8 |
| **Results** | |  |  |  |
| *Participants:* | |  |  |  |
| 14 | | When study was performed, including beginning and end dates of recruitment | Pertinent particularly to longitudinal (delayed verification) studies, authors should report recruitment dates of the study (not to be confused with recruitment dates of the wider cohort study from which it might be drawn), and the beginning (first participant) and end (last participant) dates of the periods during which index test/s and reference standard were performed.  Report the period for the index test and period for the reference standard separately if it is not clear. | 4 |
| 15 | | Clinical and demographic characteristics of the study population (at least information on age, gender, spectrum of presenting symptoms)  See also **Item 18** | Report key demographic variables: age, sex and education.  Report age distribution of sample in detail. Ethnicity and genetic factors (e.g. *APOE* genotype) may also be particularly important. The cognitive characteristics are covered in **Item 18**. | 8 |
| 16 | | The number of participants satisfying the criteria for inclusion who did or did not undergo the index tests and/or the reference standard; describe why participants failed to undergo either test (a flow diagram is strongly recommended)  See also **Item 3**, **Item 4** and **Item 5** |  | Supp. p9-11 |
| *Test results:* | |  |  |  |
| 17 | | Time-interval between the index tests and the reference standard, and any treatment administered in between | Specify the follow-up period for all subjects in relation to their outcomes. It should be specified whether or not participants had received any treatments which might affect disease progression. | 7 |
| 18 | | Distribution of severity of disease (define criteria) in those with the target condition; other diagnoses in participants without the target condition | Include a description of the severity of the target condition at the time the index test is performed. Usually captured by a cognitive score and/or duration of symptoms.  For delayed verification studies report distribution of severity of disease and the degree of certainty (such as probable/possible) about the diagnosis at time of case ascertainment.  Report other diagnoses (not target condition). Report relationship of test to other diagnoses. | 18 |
| 19 | | A cross tabulation of the results of the index tests (including indeterminate and missing results) by the results of the reference standard; for continuous results, the distribution of the test results by the results of the reference standard |  | 20-21 |
| 20 | | Any adverse events from performing the index tests or the reference standard | Report all adverse events, even if unlikely to be related to the diagnostic test performed. | N/A |
| *Estimates:* | |  |  |  |
| 21 | | Estimates of diagnostic accuracy and measures of statistical uncertainty (e.g. 95% confidence intervals)  See also **Item 12** |  | 20-21 |
| 22 | | How indeterminate results, missing data and outliers of the index tests were handled |  | Supp. p9-11 |
| 23 | | Estimates of variability of diagnostic accuracy between subgroups of participants, readers or centers, if done |  | N/A |
| 24 | | Estimates of test reproducibility, if done  See also **Item 13** |  | N/A |
| **Discussion** | | 25 |  |  |
| 25 | | Discuss the clinical applicability of the study findings | Discuss differences in age and comorbidity between the study population and the patients typically seen in clinical practice.  Discuss whether the reported data demonstrate 'added’ or ‘incremental’ value of the index test over and above other routine diagnostic tests.  Identify stage of development of the test (e.g. proof of concept; defining accuracy in a typical spectrum of patients);  Discuss the further research needed to be done to make test applicable to population in whom likely to be applied in practice. | 9-12 |

**Supplementary Material 3**

**
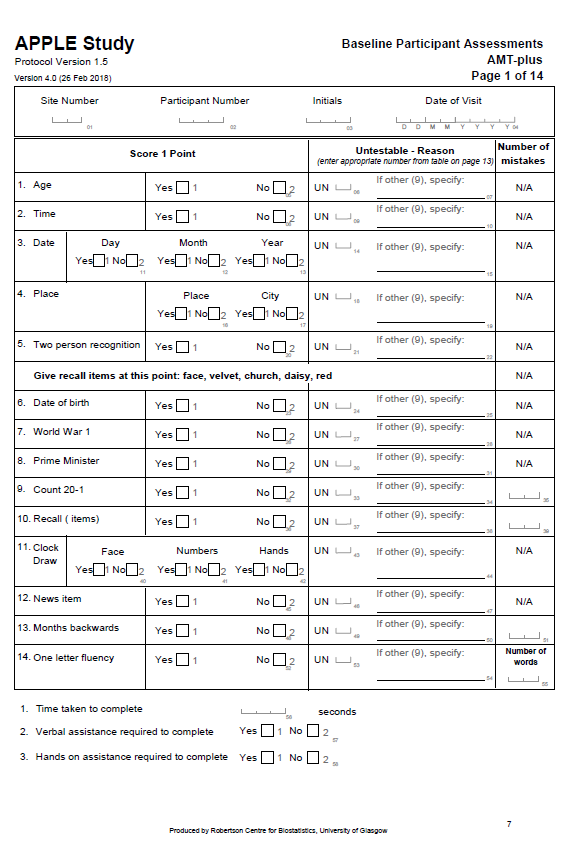
Form for baseline cognitive assessment (AMT-plus)**

**
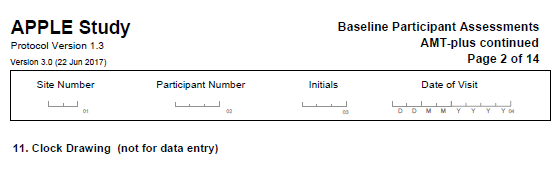
**

**Supplementary Material 4**

**
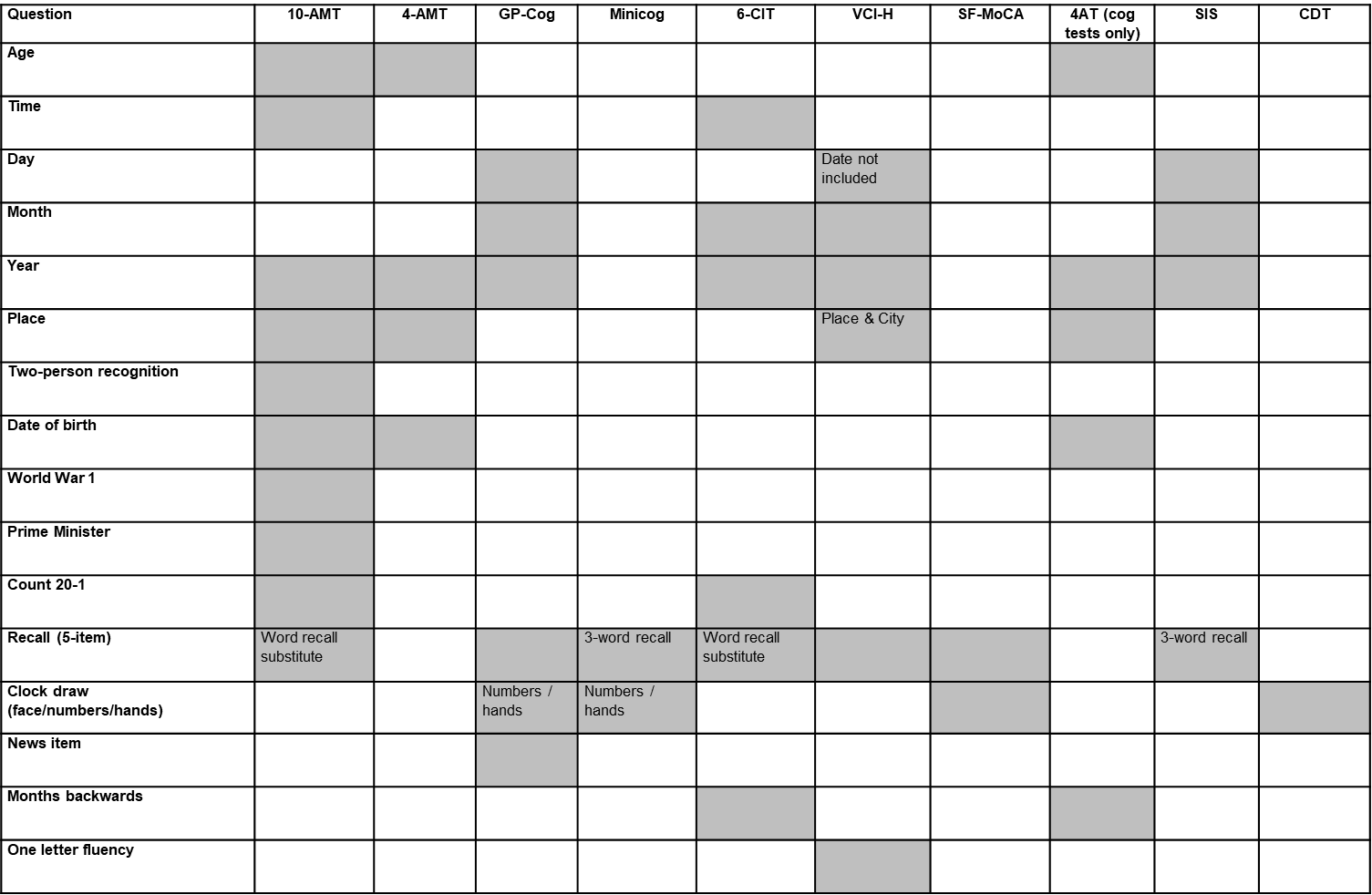
**Brief cognitive screening tests (index tests) and the items contained within them. Adapted from the APPLE protocol paper.

**Supplementary Material 5**

**Test score rules**

Rules employed and not otherwise specified in the project paper:

The recall question was standardised to 5 individual words, differing to routine tests in practice which can utilise 5- or 3-part name and addresses. For this study, if a patient successfully remembered 4 or 5 items this was taken as a score of 3/3 where 3-word recall was indicated.

Participants were not asked the date, so this was removed from the VCI-H and the total score and cut-off adjusted accordingly.

It was agreed that ‘alertness’ and ‘fluctuating course’ in the 4ATest were not relevant to the study aims and more specific to delirium. These components were removed, and the corresponding cut-off reduced.

There is no consensus on how best to score the CDT so in reference to the MoCA we decided to evaluate it out of a total of 3. This did not allow for assessment of factors such as length of clock hands.

Those who did not complete an independent question, due to for example, aphasia, received a score of 0 for that question. The same was done for those:

- drowsy/reduced consciousness
- aphasic and drowsy combined
- confused
- refused
- motor problem
- visual problem
- deaf

On the whole, these participants had the same reason code listed across the board and were therefore scored/categorised as ‘cognitively impaired’.

Two participants were listed as ‘untestable’ using multidomain neuropsychological assessments at 12 and 18 months. These participants were aphasic with missing cell data. It was assumed that these patients obtained a score of ‘0’ across the board so they were categorised as cognitively impaired, in accordance with baseline methods used.

Where there was a blank cell, this was taken as ‘0’ or ‘untestable’ for that specific question (other questions answered correctly), even though there was no corresponding reason code available to view like those above. These blank cells were few in number and are therefore unlikely to have had a large effect on results.

Where the error column contained a blank cell and the participant had got the question right, it was assumed that they made no errors and thus they scored full marks (not cognitively impaired) for that question.

For blank cells of 18-month adjudicated clinical outcomes, it was assumed that patients did not have cognitive impairment.

The 4-AMT gives one point for those who refuse to start and 2 points for those who are untestable. For the purposes of this project, we assumed that those who refused were also untestable, and they therefore received a score of ‘2’.

The reference paper used to formulate total scores for the TICS-M appointed 5 marks for ‘remote and recent memory’. Therefore, although not listed as a question in the reference paper, we included data from the question: ‘what do people usually use to cut paper?’, as the answers to this were accessible.

**Supplementary Material 6**

**Participants removed from the analysis**

**Baseline cognitive screening tests versus adjudicated 18-month neurocognitive disorder**

- One participant who had their answers described as being obtained ‘completely retrospectively – not in notes’ followed by ‘not in notes’ for the remaining questions.
- One participant who had answers to some questions before being listed as ‘discharged’.
- Two participants who had cognitive outcomes but who were missing corresponding baseline cognitive screening test scores; these participants did not undertake the baseline cognitive tests.

**Baseline cognitive screening tests versus combined 12- and 18-month multidomain neuropsychological assessments**

- Two participants who refused.
- Three participants who were too tired.
- One participant who was unwell.
- Two participants who had answered only the first couple of questions, but whose subsequent answer cells were blank; assumed that the patient gave-up, refused, or did not answer.

Note that if a patient refused an individual question during baseline assessments they were included, this differs here as it is further on in the study and the patient is refusing to complete all parts of the screening test, synonymous with withdrawing consent.

**Supplementary Material 7**

**Cross-tabulation of index test and reference standard**

Example two-by-two cross tabulation of the index test result versus adjudicated 18-month neurocognitive outcomes. This was used to calculate test accuracy metrics (sensitivity, specificity, positive and negative predictive values).

|  | | **18-Month Neurocognitive Outcome**  (Reference Standard) | |
| --- | --- | --- | --- |
|  |  | Cognitively Impaired | Not Cognitively Impaired |
| **Cognitive Screening Test**  (Index test) | Cognitively Impaired | True Positive | False Positive |
|  | Not Cognitively Impaired | False Negative | True Negative |

**Supplementary Material 8**

Table of the differences between the Areas Under the Receiver Operator Characteristic curves (AUROC) for each of the brief cognitive screening tests assessed. Results which are statistically significant are indicated by an asterisk.

| **Cognitive Screening  Test** | 10-AMT | 4-AMT | GP-Cog | Minicog | 6-CIT | VCI-H | SF-MoCA | 4AT | SIS | Informal | Cog-4 | CDT |
| --- | --- | --- | --- | --- | --- | --- | --- | --- | --- | --- | --- | --- |
| 10-AMT |  |  |  |  |  |  |  |  |  |  |  |  |
| 4-AMT | 0.03 |  |  |  |  |  |  |  |  |  |  |  |
| GP-Cog | 0.09 | 0.06 |  |  |  |  |  |  |  |  |  |  |
| Minicog | 0.06 | 0.03 | 0.03 |  |  |  |  |  |  |  |  |  |
| 6-CIT | 0.06 | 0.03 | 0.03 | 0.00 |  |  |  |  |  |  |  |  |
| VCI-H | 0.08 | 0.05 | 0.01 | 0.01 | 0.01 |  |  |  |  |  |  |  |
| SF-MoCA | 0.05 | 0.02 | 0.04 | 0.02 | 0.02 | 0.03 |  |  |  |  |  |  |
| 4AT | 0.01 | 0.03 | 0.09 | 0.07 | 0.07 | 0.08 | 0.05 |  |  |  |  |  |
| SIS | 0.10 | 0.07 | 0.01 | 0.04 | 0.04 | 0.02 | 0.05 | 0.10 |  |  |  |  |
| Informal | 0.07 | 0.10* | 0.16* | 0.13* | 0.13* | 0.14* | 0.11* | 0.06 | 0.17* |  |  |  |
| Cog-4 | 0.01 | 0.04 | 0.10 | 0.07 | 0.07 | 0.09 | 0.06 | 0.00 | 0.10* | 0.06 |  |  |
| CDT | 0.03 | 0.06 | 0.12* | 0.09 | 0.09 | 0.10* | 0.08 | 0.02 | 0.13* | 0.04 | 0.02 |  |
